# Preventing infections in non-hospital settings: long-term care.

**DOI:** 10.3201/eid0702.010210

**Published:** 2001

**Authors:** L E Nicolle

**Affiliations:** University of Manitoba, Winnipeg, Canada. lnicolle@exchange.hsc.mb.ca

## Abstract

Infection concerns in long-term care facilities include endemic infections, outbreaks, and colonization and infection with antimicrobial-drug resistant microorganisms. Infection control programs are now used in most long-term care facilities, but their impact on infections has not been rigorously evaluated. Preventive strategies need to address the changing complexity of care in these facilities, e.g., the increased use of invasive devices. The anticipated increase in the elderly population in the next several decades makes prevention of infection in long-term care facilities a priority.


### Preventing Infections in Non-Hospital Settings: Long-Term Care

**Lindsay E. Nicolle**
University of Manitoba, Winnipeg, Canada

Infection concerns in long-term care facilities include endemic infections, outbreaks, and colonization and infection with antimicrobial-drug resistant microorganisms. Infection control programs are now used in most long-term care facilities, but their impact on infections has not been rigorously evaluated. Preventive strategies need to address the changing complexity of care in these facilities, e.g., the increased use of invasive devices. The anticipated increase in the elderly population in the next several decades makes prevention of infection in long-term care facilities a priority.

In the United States, more patients are in long-term than in acute-care facilities. Long-term care facilities deliver various services to persons with a range of functional disability and disease. While some of these facilities provide care to young as well as elderly persons and psychiatric as well as medical care, most are nursing homes, which provide care to the elderly. The approach to preventing infection in nursing homes will vary with characteristics of the population.

### Infections in Long-Term Care Facilities

Infections are common in long-term care facilities (1). Major areas of concern are endemic infections, outbreaks, and colonization and infection of residents with antimicrobial-drug resistant microorganisms.

The most frequent endemic infections are respiratory tract, urinary tract, skin and soft tissue, and gastrointestinal infections (primarily manifesting as diarrhea) (Table 1). Respiratory tract infections include upper tract infections, such as pharyngitis and sinusitis, and lower tract infections, such as bronchitis and pneumonia. Pneumonia is the only infection in this setting that is often fatal (1). Urinary tract infections are the most frequent infections; while most patients are asymptomatic, the prevalence rates of bacteriuria are 25% to 50% (2). Skin and soft tissue infections include decubitus ulcers, infected vascular or diabetic foot ulcers, erysipelas, and other types of cellulitis. Nonbacterial causes of skin infection include oropharyngeal or intertriginous candidiasis, as well as herpes zoster.

Table 1. Common endemic infections in long-term care facilities (1)

| Site of infection | Frequency/1,000 patient days |
|---|---|
| Urinary tract | 0.46 – 4.4 |
| Respiratory tract | 0.1 – 2.4 |
| Skin, soft tissue | <0.1 – 2.1 |
| Gastrointestinal tract | 0 – 0.9 |

Address for correspondence: Lindsay E. Nicolle, Department of Internal Medicine, University of Manitoba, Health Sciences Centre, GG443-820 Sherbrooke Street, Winnipeg MG R3A 1R9; fax: 204-787-4826; e-mail: lnicolle@exchange.hsc.mb.ca

Many bacteria, fungi, viruses, and parasites cause outbreaks in nursing homes (Table 2). The most common are outbreaks of respiratory infection caused by influenza A (3). However, parainfluenza and respiratory syncytial viruses also cause respiratory outbreaks. Gastrointestinal outbreaks, including those caused by bacteria such as *Escherichia coli* O157:H7 and *Salmonella* species, as well as small round enteric viruses, are also common. Skin outbreaks with scabies are frequent.

Nursing home residents are at risk for colonization with antimicrobial drug-resistant microorganisms (1,4,5), including methicillin-resistant *Staphylococcus aureus* (MRSA), vancomycin-resistant enterococcus (VRE), penicillin-resistant *Streptococcus pneumoniae*, gram-negative microorganisms with extended-spectrum beta-lactamases, and increasingly, quinolone-resistant Enterobacteriaceae. Some U.S. facilities have reported rates of colonization with MRSA as high as 30% (1). Colonization with resistant microorganisms usually occurs in the acute-care facility, and transmission within the long-term care facility is uncommon in the nonoutbreak situation.

Table 2. Microorganisms reported to cause outbreaks in long-term care facilities (1)

| Viruses | Bacteria | Parasites ectoparasites |
|---|---|---|
| Influenza A,B | Group A *Streptococcus* | *Giardia lamblia* |
| Parainfluenza | *Staphylococcus aureus* | *Entamoeba histolytica* |
| Respiratory syncytial virus | *Streptococcus pneumoniae* | *Sarcoptes scabiei* |
| Caliciviruses | *Haemophilus influenzae* | |
| Adenovirus | *Bordetella pertussis* | |
| Rhinovirus | *Salmonella* spp. | |
| Coronavirus | *Shigella* spp. | |
| Rotavirus | *Campylobacter jejuni* | |
| | *Aeromonas hydrophila* | |
| | *Escherichia coli* O157:H7 | |
| | *Clostridium perfringens* | |
| | *Bacillus cereus* | |
| | *Mycobacterium tuberculosis* | |

### Considerations Unique to Long-Term Care Facilities

While the reasons for preventing infections are the same in long-term and acute-care facilities, several considerations relevant to prevention of infection differ in long-term care populations (6). For most long-term care residents, the facility is their domicile. All members of society experience infections within their homes; to what degree are unusual measures appropriate or realistic to prevent the usual infections in this setting? When is it reasonable to limit mobility or social interaction of persons in their usual residence to prevent transmission of infection?

Long-term care residents also are often highly functionally impaired. Many are incontinent, immobile, and confused or demented. The worse the functional status, the greater the likelihood of infection or colonization with resistant microorganisms (1,4,7). For example, incontinence and impaired mental status have consistently been associated with asymptomatic urinary tract infection (2). MRSA colonization is more likely to be identified in residents with pressure ulcers or fecal incontinence or who are bed bound or require feeding tubes or urinary catheters (7). In most cases, impaired functional status is a determinant of admission to long-term care and is not modifiable. If the major predictors of infection in long-term care facilities are poor functional status and co-existing chronic illness, and these conditions cannot be altered, to what extent is it realistic to anticipate that endemic infections can be prevented in such residents? In addition, with the number and severity of existing conditions, how much illness or death is attributable to infections per se, rather than to underlying chronic disease? Assessing the impact of infection on patient outcome in evaluating interventions to prevent infection is, thus, often problematic. An example is a decision to provide comfort care but not to treat pneumonia with antibiotics in severely impaired patients.

Diagnostic uncertainty is also a major issue in identifying infections and assessing interventions to prevent them. Standard clinical guidelines for surveillance of infection have been developed for long-term care facilities (8), but many barriers to diagnostic accuracy exist (9). Communication is impaired because of dementia, blindness, or deafness, and clinical assessment is complicated by symptoms associated with chronic conditions, such as cough or incontinence. The very high prevalence of asymptomatic bacteriuria means that, in a patient with nonspecific deterioration in clinical status, a positive urine culture has a low predictive value for identifying symptomatic urinary infection (10). Similarly, the high prevalence of oropharyngeal colonization with gram-negative microorganisms indicates that isolation of Enterobacteriaceae from the sputum of a person with lower respiratory tract infection has a low predictive value for identifying the infecting microorganism (2).

### Infection Control Programs

In the last 2 decades, an increasing number of long-term care facilities have developed infection control programs with surveillance and control activities (11,12). A major contribution to this development was the publication of guidelines by the Association for Professionals in Infection Control and Epidemiology (APIC) in 1991 (13). These were updated in 1997 as the Society for Healthcare Epidemiologists of America (SHEA)-APIC position paper on infection prevention and control in long-term care facilities (6). The document reviews infections in such facilities and makes specific recommendations for a feasible and relevant control program.

Differences between acute-care and long-term care facilities affect the development and management of infection control (6). Generally, long-term care facilities have fewer resources. Part-time employees or employees with many other responsibilities are often responsible for infection control, and the secretarial and computer resources may be limited. The educational level of the staff is often lower than in acute-care facilities. Radiologic and laboratory facilities are often not on site (9). Diagnostic tests may not be obtained because access to such tests requires patient transfer. Return of test results on microbiologic specimens may be prolonged. The medical record often is inadequate and access to physician resources is limited. As observation without intervention may be the more appropriate management approach in some cases, this physician shortage may lead to overuse of empiric antibiotics. Finally, limited clinical research is available to validate either an overall infection control program or specific components of a program in the long-term care facility.

SHEA-APIC infection control guidelines are evidence based (6). They categorize recommendations as A (having good evidence to support the recommendation), B (moderate evidence to support a recommendation), and C (poor evidence to support the recommendation). The quality of evidence is designated as follows: I (at least one randomized controlled trial), II (at least one well-designed clinical trial without randomization), or III (opinions of respected authorities). The infrequency of evidence designations in the guidelines demonstrates the limitations of available research (6). Only five recommendations are AI, BI, AII, or BII: for handwashing, tetanus-diphtheria immunization, annual influenza immunization, and hepatitis B and influenza immunizations for employees. All other recommendations are AIII or BIII, i.e., based on opinions of respected authorities. Thus, further evaluation of the effectiveness of specific interventions is needed.

### Clinical Trials of Interventions to Prevent Infections

Results of several recent clinical trials in long-term care settings (Table 3) have been uniformly negative with respect to the interventions assessed but are helpful in addressing the question of the extent to which endemic infections are preventable in such facilities (14-17). Many other issues relevant to specific interventions in care in long-term care facilities require assessment, particularly with the increasing use of invasive devices. For example, appropriate care needs to be explored for patients with chronic tracheostomies and respirator therapy, dialysis therapy, central lines, and percutaneous feeding tubes to limit infections and minimize cost.

### Management of Drug-Resistant Microorganisms

Antimicrobial drug-resistant microorganisms may cause illness and death in acute-care facility residents (1,4). However, it is not clear that a high prevalence of colonization with these microorganisms is associated with excess illness or death (7). In addition, no evidence supports the use of stringent barrier precautions to decrease illness or death from antimicrobial drug-resistant microorganisms in long-term care facilities (5,7). Nevertheless, such facilities have repeatedly raised barriers to admission of patients colonized

Table 3. Assessing effectiveness of selected interventions in decreasing infections in long-term care facilities

| Study question (reference) | Outcome |
|---|---|
| Does vitamin A supplementation decrease the frequency of infection? (14) | No decrease in overall occurrence of infection with vitamin A supplementation |
| Do outcomes differ with routine percutaneous feeding tube changes compared with as-needed changes? (15) | No difference in infection or other relevant outcomes with routine tube changes |
| Does treatment to eradicate MRSA[a] colonization decrease the frequency of MRSA infection? (16) | No decrease in infection with antimicrobial therapy |
| Does the frequency of symptomatic urinary infection differ with clean or sterile intermittent catheterization? (17) | No difference in frequency of infection or antimicrobial use |

[a]MRSA = methicillin-resistant *Staphylococcus aureus*.

with drug-resistant microorganisms, and management of patients colonized or infected with resistant microorganisms has sometimes been inappropriate.

Observational studies suggest that the intensity of barrier precautions, isolation or cohorting, or environmental cleaning does not decrease the likelihood of transmission of MRSA or VRE (7). Thus, additional precautions are recommended for patients colonized with these microorganisms only when the patients are a documented source of transmission to other patients (4,5) (e.g., MRSA patients with extensive skin lesions that cannot be covered or VRE patients with diarrhea and incontinence).

### Conclusions

There are many complex, unanswered questions in the prevention of infection in long-term care facilities. Priority issues for evaluation include determining the most appropriate surveillance strategies for endemic infections and identifying outbreaks early and efficiently. Recommendations for influenza A have been made (3). However, when should cultures be obtained from patients with diarrhea? What is appropriate surveillance for endemic infections, and should it be focused only in areas where an opportunity for prevention exists?

The feasibility of preventing endemic infections requires further study. In addition, the feasibility of decreasing or preventing high colonization rates with drug-resistant microorganisms in long-term care facility residents needs to be assessed, since most patients acquire these microorganisms in acute-care facilities. Practices related to antimicrobial-drug use are key to this question. In addition to controlled comparative trials to identify appropriate antimicrobial-drug use, patients who do not require treatment need to be identified. The role of drug therapy in preventing infections is also not adequately studied. Finally, an infection control program may be costly. What are the benefits of such a program? Decreased length of stay, for example, will not usually be a meaningful outcome. Thus, while substantial progress has been made in the past decade in managing infection prevention, many issues still need to be answered. As the elderly population will increase in the next two decades, addressing these problems must be a priority.

Dr. Nicolle is a professor in the Department of Internal Medicine, University of Manitoba. Her research interests include urinary tract infection, infection in the elderly, and antimicrobial-drug resistant organisms in health-care facilities.

### References

1. Nicolle LE, Strausbaugh LJ, Garibaldi RA. Infections and antibiotic resistance in nursing homes. Clin Microbiol Rev 1996;9:1-17.
2. Nicolle LE. Asymptomatic bacteriuria in the elderly. Infect Dis Clin North Am 1997;11:647-62.
3. Bradley SF. Long-term Care Committee of the Society for Health Care Epidemiology of America. Prevention of influenza in long-term care facilities. Infect Control Hosp Epidemiol 1999;20:629-37.
4. Strausbaugh LJ, Crossley KB, Nurse BA, Thrupp LD, SHEA Long-term Care Committee. Antimicrobial resistance in long-term care facilities. Infect Control Hosp Epidemiol 1995; 17:120-9.
5. Crossley K. Long-term Care Committee of the Society for Health Care Epidemiology of America. Vancomycin-resistant enterococci in long-term care facilities. Infect Control Hosp Epidemiol 1998;19:521-5.
6. Smith PW, Rusnak PG. Infection prevention and control in the long-term care facility. Infect Control Hosp Epidemiol 1997;18:831-49.
7. Bradley S. Issues in the management of resistant bacteria in long-term care facilities. Infect Control Hosp Epidemiol 1999;20:362-6.
8. McGeer AR, Campbell B, Emori TG, Heirholzer WJ, Jackson MM, Nicolle LE, et al. Definitions of infection for surveillance in long-term care facilities. Am J Infect Control 1991;19:1-7.
9. Nicolle LE, Bentley D, Garibaldi R, Neuhaus E, Smith P, SHEA Long-term Care Committee. Antimicrobial use in long-term care facilities. Infect Control Hosp Epidemiol 1996;17:119-28.
10. Orr P, Nicolle LE, Duckworth H, Brunka J, Kennedy J, Murray D, et al. Febrile urinary infection in the institutionalized elderly. Am J Med 1996;100:71-7.
11. Goldrick BA. Infection control programs in skilled nursing long-term care facilities: An assessment, 1995. Am J Infect Control 1997;27:4-9.
12. Smith PW. Development of nursing home infection control. Infect Control Hosp Epidemiol 1999;20:303-5.
13. Smith PW, Rusnak PG. Guideline for infection prevention and control in the long-term care facility. Am J Infect Control 1991;19:198-215.
14. Murphy S, West KP, Greenough WB, Cherot E, Katz J, Clement L. Impact of vitamin A supplementation on the incidence of infection in elderly nursing home residents: A randomized controlled trial. Age Ageing 1992;21:435-9.
15. Graham S, McIntyre M, Chicoine J, Gerard B, Laughren R, Cowley G, et al. Frequency of changing enteral alimentation bags and tubing and adverse clinical outcomes in patients of a long-term care facility. Can J Infect Control 1993;8:41-3.
16. Strausbaugh LJ, Jacobson C, Sewell DL, Potter S, Ward TT. Antimicrobial therapy for methicillin-resistant *Staphylococcus aureus* colonization in residents and staff of a Veterans Affairs nursing home care unit. Infect Control Hosp Epidemiol 1992;13:151-9.
17. Duffy LM, Cleary J, Ahern S, Kushowski MA, West M, Wheeler L, et al. Clean intermittent catheterization: Safe, cost-effective bladder management for male residents of VA nursing homes. J Am Geriatr Soc 1995;43:865-70.

## References

[PDF_00281] Bradley S. F. (1999). Issues in the management of resistant bacteria in long-term-care facilities.. Infect Control Hosp Epidemiol.

[PDF_00269] Bradley S. F. (1999). Prevention of influenza in long-term-care facilities. Long-Term-Care Committee of the Society for Healthcare Epidemiology of America.. Infect Control Hosp Epidemiol.

[PDF_00313] Duffy L. M., Cleary J., Ahern S., Kuskowski M. A., West M., Wheeler L., Mortimer J. A. (1995). Clean intermittent catheterization: safe, cost-effective bladder management for male residents of VA nursing homes.. J Am Geriatr Soc.

[PDF_00292] Goldrick B. A. (1999). Infection control programs in skilled nursing long-term care facilities: an assessment, 1995.. Am J Infect Control.

[PDF_00304] Graham S., McIntyre M., Chicoine J., Gerard B., Laughren R., Cowley G., Morrison J., Aoki F. Y., Nicolle L. E. (1993). Frequency of changing enteral alimentation bags and tubing, and adverse clinical outcomes in patients in a long term care facility.. Can J Infect Control.

[PDF_00283] McGeer A., Campbell B., Emori T. G., Hierholzer W. J., Jackson M. M., Nicolle L. E., Peppler C., Rivera A., Schollenberger D. G., Simor A. E. (1991). Definitions of infection for surveillance in long-term care facilities.. Am J Infect Control.

[PDF_00300] Murphy S., West K. P., Greenough W. B., Cherot E., Katz J., Clement L. (1992). Impact of vitamin A supplementation on the incidence of infection in elderly nursing-home residents: a randomized controlled trial.. Age Ageing.

[PDF_00267] Nicolle L. E. (1997). Asymptomatic bacteriuria in the elderly.. Infect Dis Clin North Am.

[PDF_00286] Nicolle L. E., Bentley D., Garibaldi R., Neuhaus E., Smith P. (1996). Antimicrobial use in long-term-care facilities.. Infect Control Hosp Epidemiol.

[PDF_00264] Nicolle L. E., Strausbaugh L. J., Garibaldi R. A. (1996). Infections and antibiotic resistance in nursing homes.. Clin Microbiol Rev.

[PDF_00289] Orr P. H., Nicolle L. E., Duckworth H., Brunka J., Kennedy J., Murray D., Harding G. K. (1996). Febrile urinary infection in the institutionalized elderly.. Am J Med.

[PDF_00295] Smith P. W. (1999). Development of nursing home infection control.. Infect Control Hosp Epidemiol.

[PDF_00297] Smith P. W., Rusnak P. G. (1991). APIC guideline for infection prevention and control in the long-term care facility.. Am J Infect Control.

[PDF_00279] Smith P. W., Rusnak P. G. (1997). Infection prevention and control in the long-term-care facility. SHEA Long-Term-Care Committee and APIC Guidelines Committee.. Infect Control Hosp Epidemiol.

[PDF_00308] Strausbaugh L. J., Jacobson C., Sewell D. L., Potter S., Ward T. T. (1992). Antimicrobial therapy for methicillin-resistant Staphylococcus aureus colonization in residents and staff of a Veterans Affairs nursing home care unit.. Infect Control Hosp Epidemiol.

